# Response Surface Optimization of Extraction Conditions and In Vitro Antioxidant and Antidiabetic Evaluation of an Under-Valued Medicinal Weed, *Mimosa pudica*

**DOI:** 10.3390/plants10081692

**Published:** 2021-08-18

**Authors:** Nor Saffana Baharuddin, Muhamad Aidilfitri Mohamad Roslan, Mohsen Ahmed Mohammed Bawzer, Azzreena Mohamad Azzeme, Zuraida Ab Rahman, Mohd Ezuan Khayat, Nor Aini Abdul Rahman, Zulfazli M. Sobri

**Affiliations:** 1 Department of Bioprocess Technology, Faculty of Biotechnology and Biomolecular Sciences, Universiti Putra Malaysia, Serdang 43400, Selangor, Malaysia; nsaffana@gmail.com (N.S.B.); fitree91@gmail.com (M.A.M.R.); mohsenbaw2@gmail.com (M.A.M.B.); nor_aini@upm.edu.my (N.A.A.R.); 2 Department of Biochemistry, Faculty of Biotechnology and Biomolecular Sciences, Universiti Putra Malaysia, Serdang 43400, Selangor, Malaysia; azzreena@upm.edu.my (A.M.A.); m_ezuan@upm.edu.my (M.E.K.); 3 Biotechnology Research Centre, MARDI Headquarters, Persiaran MARDI-UPM, Serdang 43400, Selangor, Malaysia; azuraida@mardi.gov.my; 4 Bioprocessing and Biomanufacturing Research Centre, Faculty of Biotechnology and Biomolecular Sciences, Universiti Putra Malaysia, Serdang 43400, Selangor, Malaysia

**Keywords:** *Mimosa pudica*, antioxidant, antidiabetic, 3T3-L1, response surface methodology

## Abstract

*Mimosa pudica* Linn is a well-known perennial herb and is traditionally used in ayurvedic medicine for the treatment of various illnesses. Despite its abundance in nature, the therapeutic potential of this invasive weed is deemed to be underappreciated in Malaysia. Previous studies have found an abundance of bioactive compounds associated with potent antioxidant properties in all parts of the plant. However, the optimum parameters required for the extraction of antioxidant compounds are still unknown. Therefore, the present study aimed to optimize the solvent extraction parameters of *M. pudica* using response surface methodology to enrich the accumulation of antioxidant compounds in the extracts. The effects of the optimized *M. pudica* extracts were then evaluated on the cell viability and glucose uptake ability in a 3T3-L1 adipocyte cell line. The highest total phenolic (91.98 mg of gallic acid equivalent per g of the dry extract) and total flavonoid content (606.31 mg of quercetin equivalent per g of the dry extract) were recorded when using 100% ethanol that was five-fold and three-fold higher, respectively, as compared to using 50% ethanol. The extract concentration required to achieve 50% of antioxidant activity (IC_50_ value) was 42.0 µg/mL using 100% ethanol as compared to 975.03 µg/mL using 50% ethanol. The results indicated that the use of 100% ethanol solvent had the greatest impact on the accumulation of antioxidant compounds in the extract (*p* < 0.05). Cell viability assay revealed that all extract concentration treatments recorded a viability level of above 50%. Glucose uptake assay using 2-NBDG analog showed that the cells treated with 50 µg/mL extract combined with insulin were five-fold higher than the control group. Given the high antioxidant and antidiabetic properties of this plant, *M. pudica* can be easily highlighted as a plant subject of interest, which warrants further investigation for nutraceutical prospects.

## 1. Introduction

*Mimosa pudica* Linn. is a perennial herb species that belongs to the family of *Fabaceae*. It is commonly known as sensitive plant or shy plant and recognized for its shrinking reaction to contact as a defense mechanism. The whole parts of the plant are incorporated as medicine in the Ayurveda, Indian traditional medicine system. Due to its therapeutic values, it has been used in ethnomedicine for centuries in treating various diseases, such as diabetes, fever, wounds, ulcers, and dyspepsia [1]. This creeping perennial shrub is originated from tropical climate regions like Southeast Asia and can be easily found on roadsides, walkways, croplands, and home gardens in countries like Malaysia, India, China, Bangladesh, Philippines, etc. This plant species is easily dismissed as weeds owing to its invasive behavior, which causes inconveniences in home gardens and cropland maintenance.

Previous phytochemical investigations on *M. pudica* have resulted in the identification of at least 40 well-known chemical constituents from the group of alkaloids, phenols, and flavonoids. Through high-performance liquid chromatography (HPLC), gas chromatography-mass spectrometry (GC-MS), and liquid chromatography-mass spectrometry (LC-MS) techniques, high profusion bioactive compounds of *M. pudica* have been detected with a good range of recovery and detection limit [2,3,4,5]. Recent phytochemical profiling of *M. pudica* by Ahuchaogu et al. [6] revealed the presence of flavonoids C-glycosides, sterols, terpenoids, tannins, fatty acids, ascorbic acid, crocetin, D-glucuronic acid, linoleic acid, palmitic acid, stearic acid, mimosine, D-xylose, and *β*-sitosterols. Several secondary metabolites of *M. pudica* have been successfully isolated, which include bufadienolide, D-pinitol, norepinephrine, P-coumaric acid, mimopudine, and mimosine. Additionally, its crude extracts and isolates have been reported to exhibit a broad spectrum of in vitro and in vivo pharmacological activities, including antidiabetic, antimicrobial, wound healing, antidepressant, and ethnoveterinary potential, among others [7]. Despite its abundance and wide-ranging medicinal properties, it is deemed to be underappreciated and perceived as an undesirable plant.

Diabetes mellitus is a chronic metabolic disease marked by hyperglycemia, or elevated blood glucose levels, caused by insulin deficiency (type 1 diabetes) or by insulin resistance (type 2 diabetes) [8,9,10]. Global statistics have recorded that 463 million individuals worldwide were diagnosed with diabetes within the year 2018 to 2019, where 3.6 million among them were Malaysians [11]. The most prevalent kind of diabetes is type 2 diabetes mellitus, which is characterized by the imbalance between insulin production and blood sugar absorption, resulting in a greater risk of both acute and long-term complications, as well as mortality at a younger age. Since type 2 diabetes is considered avoidable and treatable, many researchers have been focusing on its management and alternative treatments [11]. 

A substantial amount of data suggests that hyperglycemia is the primary cause of oxidative stress, which is linked to a fundamental step in the onset and progression of diabetes problems [12,13]. This highlights the correlation between oxidative stress and diabetes, even though the exact processes by which oxidative stress increases the development of diabetes complications are only partially understood [14,15]. Hyperglycemia-induced oxidative stress, which results in the creation of free radical species, stimulates the synthesis of inflammatory mediators [16]. Free radicals are unstable kinds of molecules that target healthy cells to couple up their odd free electrons, which results in cellular dysfunction [17]. The occurrence of reactive oxygen species (ROS) has been linked to various diseases or disorders like cancers, diabetes, hyperuricemia, gout, and inflammatory diseases [18]. ROS or free radicals found in the human biological systems can damage various molecules, such as DNA, and eventually inhibit cell functions. Antioxidants are a class of chemical compounds that actively or indirectly decrease free radicals and prevent oxidation. Plant-based antioxidants are free radical scavenging agents that can reduce the harmful effects of unstable species on the human body and may be effective in the treatment of diabetic problems [19].

Exogenous supplementation of antioxidants can relieve oxidative stress in the body. However, some synthetic antioxidants have demonstrated possible harmful effects, such as liver damage and carcinogenesis, in the long term [20,21,22]. Academics and pharmaceutical institutions have been racing in a search for novel secondary metabolites in plants, with the hope of discovering new approaches in the treatments of diseases [23]. 

The most common conventional optimization techniques include the one-factor-at-a-time (OFAT) or the single-factor experiment techniques. OFAT is considered a laborious and cost-inefficient approach since a multitude of runs of experiments is required to achieve a consensus. Conversely, response surface methodology (RSM) is a powerful multivariable optimization technique, which is generally easier and more statistically robust [24]. This tool describes the interactional effects between a set of data and can be used to generate models for the prediction of the responses [25]. Previous RSM-based optimization of *M. pudica* extraction methods used methanol as the sole solvent while incorporating microwave-assisted extraction to obtain maximum antioxidative compounds [26]. Although the results seemed promising, other solvents, such as ethanol, have yet to be tuned using the same optimization method.

In light of the potentially high nutraceutical values of *M. pudica*, the present study aimed to optimize the extraction conditions of *M. pudica* to enrich the accumulation of antioxidant compounds through the Box-Behnken Design of RSM, and thus evaluate the antioxidant and antidiabetic potential of the optimized crude extract.

## 2. Materials and Methods

### 2.1. Box-Behnken Design

The Box-Behnken design (BBD) was used to obtain the experimental design, analysis of results, and regression models. An experimental design using BBD was established using three factors (extraction time, temperature, ethanol concentration) with three levels (30–90 min, 40–80 °C, 50–100% ethanol) according to the range of parameters described in previous reports [27,28,29]. The effects of extraction time (A), water bath temperature (B), and ethanol concentration (diluted with water) (C) on the response, i.e., crude extract yield, IC_50_ value, total phenolic content (TPC), and total flavonoid content (TFC), were investigated to determine the optimal extraction parameters. The entire design consisted of 17 experimental runs, which were carried out in a random sequence. 

### 2.2. Preparation of Plant Extracts 

The air-dried aerial parts of *M. pudica* were purchased from a local farmer in Changloon, Kedah. The sample was pulverized using a commercial blender (PB-3202L, Panasonic, China), sieved, and stored in a sealed container at room temperature for future use. The extracts were prepared following [30] with minor modifications. Solvent extraction was conducted by mixing 10 g of the powdered sample with 50 mL of ethanol (Avantor, Radnor, PA, USA) of varying concentrations (50%, 75%, and 100%), and left at different extraction times and temperature levels in a water bath (WB-11, WiseBath, Brighton, CO, USA). The solvent from primary extracts was evaporated under a reduced pressure using a rotary evaporator (Rotary Evaporator N-1000, Eyela Oilbath OSB-2000, and Eyela N-1000 Aspirator, Tokyo, Japan) and the resulting filtrate was diluted to 2 mg/mL in absolute ethanol to yield a stock solution. The stock solution was further diluted to between 0.05 and 1.00 mg/mL as the working sample solution. The extraction yield was calculated using the following Equation (1) by [31]:

Y = Wd/Ve × Rss × 100(1)

where Wd is the weight of dried extract (g), Ve is the volume of aqueous filtered (mL), and Rss is the ratio of solvent to solid (mL/g).

### 2.3. Determination of DPPH Radical Scavenging Assay 

The antioxidant activity of the extracts against DPPH radicals was evaluated according to the method previously described by Patro et al. [32] with slight modifications. Exactly 0.5 mL of each sample with a concentration between 0.05 and 1.00 mg/mL were mixed with 1.0 mL of 0.1 mM DPPH reagent (Sigma Aldrich, St. Louis, MO, USA). The reaction mixture was incubated in the dark at room temperature for 30 min. The absorbance of the reaction was then recorded at 517 nm using a UV-visible spectrophotometer (SQ2800, Unico, Dayton, NJ, USA). The antioxidant efficacy of all samples was compared to ascorbic acid at 25–200 µg/mL (Loba Chemie, Mumbai, India) as a positive control in triplicate using the following Equation (2):

DPPH radical scavenging activity (%) = [(C − S)/C] × 100(2)

where C is the absorbance value of the control and S is the absorbance value of the sample.

### 2.4. Determination of Total Phenolic Content (TPC) 

The TPC of the extracts was measured according to the method reported by Ibrahim et al. [33] with slight modifications. Briefly, samples of 0.1 mg/mL were mixed with 1.0 mL of Folin-Ciocalteu reagent (Sigma Aldrich, St. Louis, MO, USA) and shaken for 5 min before 10 mL of 7% Na_2_CO_3_ (Himedia Labs, Mumbai, India) was added. The mixture solutions were adjusted with ethanol to a volume of 25 mL, mixed thoroughly, and incubated at ambient temperature in dark conditions. After 90 min, the absorbance was measured at 750 nm. The TPC was expressed as milligrams of gallic acid (Sigma Aldrich, St. Louis, MO, USA) equivalent per gram of extract (mg GAE/g extract) using the calibration curve equation: y = 0.0066x + 0.0379, R^2^ = 0.9975, at concentrations between 50 and 500 mg/L.

### 2.5. Determination of Total Flavonoid Content (TFC) 

The TFC of the extracts was measured as previously described by Chandra et al. [34] with slight modifications. Exactly 500 µL of the sample with a concentration of 1 mg/mL was mixed with 150 µL of 5% NaNO_3_ (R&M Chemical, Subang, Selangor) and left for 5 min. Then, 150 µL of 10% Al_2_Cl_3_ (R&M Chemical, Subang, Selangor) was added into the mixture and left for 6 min. About 1.0 mL of 1.0 M NaOH (R&M Chemical, Subang, Selangor) was added and the mixture was vortexed. The absorbance was measured at 510 nm. TFC was calculated using the calibration curve of quercetin (Sigma Aldrich, USA) equation: y = 0.0008x + 0.1909, R^2^ = 0.9793, at concentrations between 20 and 200 µg/mL and expressed as mg of quercetin equivalent per g of extract (mg QE/g extract). 

### 2.6. Cell Passaging and Maintenance 

Complete growth media (CGM) was prepared by combining 89% Dulbecco’s Modified Eagle’s medium (DMEM) (Nacalai Tesque, Kyoto, Japan) high glucose with 10% fetal bovine serum (FBS) (Nacalai Tesque, Kyoto, Japan) and 1% penicillin-streptomycin (Nacalai Tesque, Kyoto, Japan). The 3T3-L1 cell line was obtained from American Type Culture Collection (ATCC, Manassas, VA, USA). The cell line was routinely trypsinized into a fresh CGM when it reached 70–80% confluency. The media was removed from the flask using a 5 mL serological pipette. The cells left in the flask were washed with 5 mL Dulbecco’s Phosphate Buffered Saline (D-PBS) (Sigma Aldrich, St. Louis, MO, USA). Exactly 2 mL of trypsin (Nacalai Tesque, Kyoto, Japan) was added to detach the cells from the flask wall. The culture was incubated for 5 min to let the trypsin act. The flask was then tapped gently and observed under a microscope (Primostar 3, Zeiss, Germany) to ensure cell detachment. About 4 mL of CGM were added to neutralize the trypsin. The cells were dislodged from the walls gently a few times. The mixture was transferred into a new 15 mL falcon tube. An appropriate volume of culture media was calculated to culture the cells. A sufficient volume of media was added and seeded into a new culture flask of approximately 6 × 10^5^–7 × 10^5^ cells per flask. The cells were spread evenly and incubated at 37 °C with 5% CO_2_ in a humidified incubator and maintained in a sub-confluent state until further use.

### 2.7. Cell Viability Assay (MTT Assay) 

Cell viability was determined by the 3-(4,5-dimethylthiazol-2-yl)-2,5-diphenyltetrazolium bromide (MTT) (Sigma Aldrich, St. Louis, MO, USA) assay as described by [10]. Cells at 80% of confluency were seeded into a 96-well clear plate and incubated for 48 h. Then, the cells were treated with extract compounds in serial dilution ranging from 50–800 µg/mL in triplicate following incubation for another 48 h. The media were aspirated, and the wells were washed with 100 µL of D-PBS. Subsequently, 100 μL of DMEM without glucose were added along with 100 µL of 0.5 mg/mL MTT reagent followed by a 4-h incubation. The violet-formazan crystals were dissolved in 100 µL of dimethyl sulfoxide (DMSO) (Nacalai Tesque, Kyoto, Japan). The absorbance was measured at 570 nm using a microplate reader (MR9600, Accuris, Sayreville, NJ, USA) and the percentage viability of the cells was calculated using the following formula (3):

% viable cells = [(abs sample − abs blank)/(abs control − abs blank)] × 100(3)

### 2.8. Differentiation of 3T3-L1 Cells into Mature Adipocytes 

Differentiation was induced by seeding cells at 70–80% confluency into a 96-well black with clear-bottom plate (Greiner Bio-One, Frickenhausen, Germany) and grown to confluence to initiate the growth arrest phase. Then, the media were changed to induction media containing DMEM high glucose supplemented with an adipogenic cocktail (2.5 µL insulin, 1 µL DEXA, 10 µL IBMX for every mL of DMEM) (Nacalai Tesque, Kyoto, Japan). After two days, the media were replaced with DMEM high glucose supplemented with insulin only. Subsequently, the media were changed every 48 h with fresh CGM. After 7–10 days, fully differentiated adipocytes were obtained. 

### 2.9. Glucose Uptake Assay 

The differentiated adipocytes were cultured with glucose-free DMEM. Following 3 h of incubation, the media were aspirated and rinsed with 100 µL of 1.0 M KRPH buffer (20 mM HEPES, 137 mM NaCl, 4.7 mM KCl, 1.2 mM MgSO_4_, 1.2 mM KH_2_PO_4_, 2.5 mM CaCl_2_, and 2 mM pyruvate; pH 7.4). Then, the cells were treated with 100 µL of blank, control, and experimental compounds at the indicated concentrations followed by 10 min of incubation. Exactly 100 µL of 150 µg/mL 2-NBDG (Cayman Chemical, Ann Arbor, MI, USA) were added to each well and incubated for another 30 min. The plate was centrifuged for 5 min at 400× *g* at room temperature and the supernatant was aspirated. Then, 200 µL of cell-based assay buffer were added to each well. Extra care was applied to not disturb the cell layer. The plate was centrifuged again, the supernatant was removed, and 100 µL of cell-based assay buffer were added to each well. The cells were ready for analysis and analyzed immediately. The 2-NBDG taken up by cells was determined at an excitation wavelength of 475 nm and an emission wavelength of 550 nm using a multi-mode microplate reader (M5, SpectraMax, San Jose, CA, USA).

### 2.10. Statistical Analysis 

The BBD in RSM was performed using Design Expert Software (Version 11.0, State-Ease Inc., Minneapolis, MIN, USA). ANOVA was used to summarize the results obtained under all the experimental conditions. The confidence interval of 95% was set to test the significant effect of the factors and their interaction. The *F* statistic test was used to evaluate whether the regression model was adequate to describe the observed data. The percentage of variability of the optimization parameter was analyzed by *R* squared statistics. The optimal extraction conditions were estimated through regression analysis and 3-D response surface plots. A confirmation experimental run was conducted to verify the validity of the statistical experimental strategies. The cell viability (MTT) assay and glucose uptake assay were analyzed by one-way ANOVA, and the Tukey post hoc test was used for the statistical significance declaration. Both were analyzed using SPSS version 23 (IBM, Armonk, NY, USA), where *p* < 0.05 was considered significant.

## 3. Results and Discussion

### 3.1. Optimization of Extraction Conditions

The experimental conditions and results are shown in Table 1, and the ANOVA results are exhibited in Table 2. Multiple regressions were used to analyze the data and determine the relative relationship between the test variable and the response variable, which was defined by second-order polynomial Equations (4)–(7), respectively:

Extract yield, Y_1_ = 16.16 + (−0.7875A) + 0.5125B + (−7.2C) + 1.6AB + 1.775AC + 1.575BC + 1.495A^2^ + 0.145B^2^ + (−4.93C^2^) (4)

IC_50_ value, Y_2_ = 126.274 + (−42.3637A) + 126.288B + (−311.681C) + 33.6525AC + (−116.5BC) + 180.382A^2^ + (−50.2308B^2^) + 172.957C^2^ (5)

TPC, Y_3_ = 57.30 + 9.39A + 0.6700B + 28.44C + (−6.70AB) + (−0.2300AC) + 3.27BC + (−0.6490A^2^) + 2.49 B^2^ + (−3.17C^2^) (6)

TFC, Y_4_ = 397.89 + 9.22A − 0.9387B + 149.22C + (−65.47AB) + (−9.22AC) + (−49.84BC) + (−61.05A^2^) + 62.70C^2^ (7)

where A, B, and C represent the extraction time, temperature, and ethanol concentration, respectively. 

#### 3.1.1. Extract Yield

The extract yield of *M. pudica* ranged from 3.1% to 25.9%. The highest yield (25.9%) was observed in experimental run 2 under extraction conditions of 30 min extraction time, 60 °C temperature, and ethanol concentration of 50%. This extraction protocol resulted in a higher yield as compared to a previous study on *M. pudica* extraction, where only 20% extract yield was obtained using 80% ethanol as solvent, at room temperature, and an extraction time of 3 days [35]. Based on the ANOVA results in Table 2, only the linear (A) and the quadratic (A^2^) response of the ethanol concentration were highly significant (*p* < 0.05) while the extraction time, temperature, and the variable interactions were less significant (*p* > 0.05).

**Figure 1:**
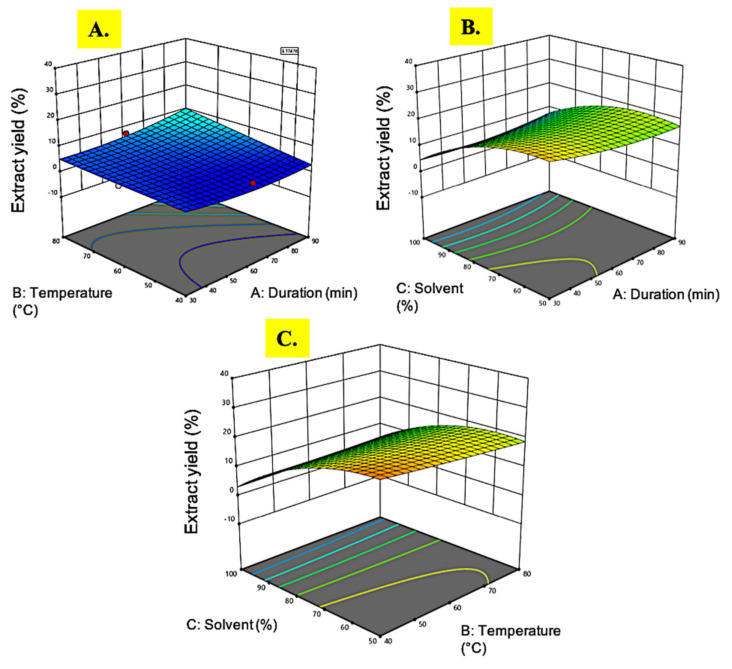
Response surface analysis (3D) of *M. pudica* extract on crude extract yield (**A**) effect of temperature and extraction time; (**B**) effect of extraction time and ethanol concentration; (**C**) effect of temperature and ethanol concentration.

As the solvent concentration of ethanol decreased from 100% to 50%, the extract yield increased, as portrayed in Figure 1B. The stronger polar strength of diluted ethanol (water as a diluent) as compared to absolute ethanol might explain the reason behind the higher yield of the crude extract [36]. The outcome of this study revealed a similar pattern with a previous study by Nawaz et al. [37] on the extraction of *Phaseolus vulgaris*, where the stronger polarity of the solvent resulted in a higher extract yield. The interaction between the solvent and temperature showed a notable effect to increase the extract yield (Figure 1C) since the highest reading was achieved when both the ethanol concentration and temperature were at the lowest levels. On the contrary, earlier reports showed that an increase in temperature could steer up the analyte solubility by the mass transfer rate since it leads to a decrease in surface tension and viscosity, which aids the solvent to reach sample matrices, hence improving the extraction rate [38]. This inconsistency might be due to the intervention of the solvent reaction, which obviously play a significant role in determining plant extraction efficacy apart from the temperature alone.

#### 3.1.2. IC_50_ Value

The IC_50_ value (half maximal inhibitory concentration) is the concentration of the sample that can scavenge 50% of DPPH free radical in a DPPH free radical scavenging assay. The IC_50_ value is inversely proportional to the free radical scavenging activity or the antioxidant property of the sample [39]. A lower IC_50_ value indicates a higher antioxidant property. The IC_50_ value from *M. pudica* extracts ranged from 42.0 µg/mL to 975.03 µg/mL. The lowest IC_50_ value was observed in experimental run 3 under the extraction conditions of 60 min at 40 °C in 100% ethanol. The ANOVA of the regression coefficient revealed that the two linear parameters, temperature (B) and ethanol concentration (C), were significant (*p* < 0.05) along with the quadratic effects of extraction time (A^2^) and ethanol concentration (C^2^), which had the greatest impact on the IC_50_ values (Table 2).

**Figure 2:**
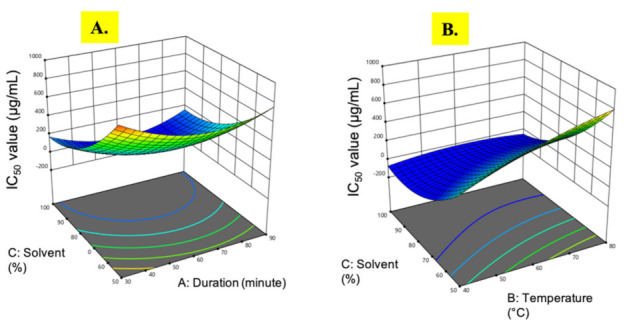
Response surface analysis (3D) of *M. pudica* extract on the IC_50_ value (**A**) effect of extraction time and ethanol concentration; (**B**) effect of temperature and ethanol concentration.

Based on Figure 2A, when the 100% ethanol concentration was used, the IC_50_ value fluctuated and maintained below 200 µg/mL as the extraction time increased. However, the temperature change did not give any effects on the IC_50_ value (Figure 2B). Notably, at the 50% ethanol concentration, an increase in temperature gave a negative effect on the IC_50_ value, where the reading increased to nearly 700 µg/mL. Contrary to a previous report on *Barleria lupulina* extracts [40], free radical scavenging activity was reduced with increasing ethanol concentration, while it was significantly increased with a prolonged extraction time. Although a longer extraction time might increase the quantity of extracted analytes [41], there is a possibility that the extracted compounds are degraded during the process. Therefore, there is a need to strike a balance among the extraction conditions to find the optimal values necessary to achieve the maximal IC_50_ value.

#### 3.1.3. Total Phenolic Content

TPC was measured using gallic acid equivalent quantification. The TPC in the *M. pudica* extract ranged from 14.51 to 91.98 mg GAE/g. The highest TPC (91.98 mg GAE/g) was observed in experimental run 10 with the extract conditions of 90 min at 60 °C in a 100% ethanol concentration. The results observed in this investigation were far higher than those observed by [42], where the highest TPC in *M. pudica* extract was only 60.07 mg GAE/g obtained through unoptimized solvent extraction. However, a study conducted by Ganesan et al. [26] using microwave-assisted extraction on *M. pudica* obtained a much higher recovery of TPC up to 640.0 mg GAE/g. This discrepancy could be attributed to the rapid heating of microwave irradiation, which promotes cellular matrix breakdown and increases phenolic compound release [43]. Based on Table 2, the regression coefficient showed that only the ethanol concentration (C) was highly significant on the TPC (*p* < 0.05) while all the other quadratic and interaction terms showed no notable effects (*p* > 0.05).

**Figure 3:**
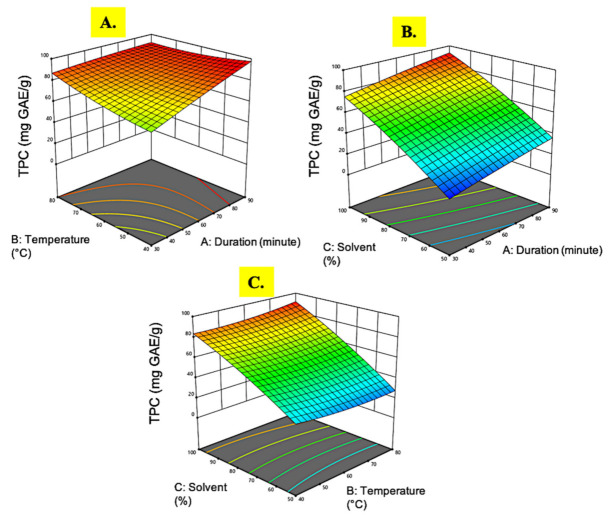
Response surface analysis (3D) of *M. pudica* extract on TPC (**A**) effect of extraction time and temperature; (**B**) effect of extraction time and ethanol concentration; (**C**) effect of temperature and ethanol concentration.

The 3D plot graphs in Figure 3 demonstrates that the ethanol concentration was a single significant factor influencing the TPC value. In Figure 3A, it can be seen that the TPC value decreased marginally even at the lowest extraction time and temperature level. A previous study on wheat species extraction conformed the current finding that the extraction time did not have a significant role in TPC accumulation in plant extracts [44]. On the contrary, Guido and Moreira [45] reported that extraction temperature was important for phenols extraction purposes. At higher temperatures, the compounds’ solubility increases while the surface tension decreases, resulting in weaker phenolic–protein and phenolic–polysaccharide linkages, hence easing the movement of phenolic compounds into the extraction solvent [46]. However, it was found that high extraction temperatures negatively affect the phenol extraction of brewer’s spent grain, as described by Ogbole et al. [47] and Mousinho et al. [48]. Therefore, the effects of temperature on phenolic compound extraction are still not fully understood and are rather plant species dependent.

#### 3.1.4. Total Flavonoid Content

**Figure 4:**
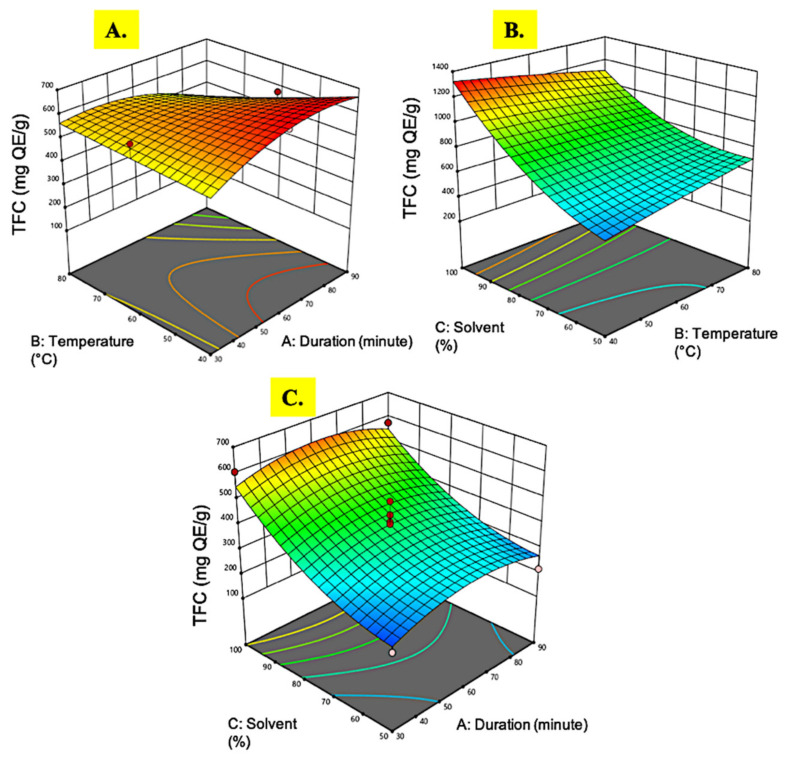
Response surface analysis (3-D) of *M. pudica* extract on TFC (**A**) effect of extraction time and temperature; (**B**) effect of temperature and ethanol concentration; (**C**) effect of extraction time and ethanol concentration.

Flavonoids are a family of natural phenolic chemicals produced as bioactive secondary metabolites in plants and are responsible for taste, color, and pharmacological properties. They are powerful antioxidants with therapeutic values, such as anti-inflammatory and anticancer [49]. The TFC in *M. pudica* extracts in this study was measured using quercetin equivalent quantification. The TFC values recovered from *M. pudica* extracts ranged from 204.44 to 606.31 mg QE/g. The ANOVA results in Table 2 and the 3D plot in Figure 4 showed a similar pattern as in the TPC recovery. The extraction conditions employed in this study resulted in an exceptional amount of flavonoids accumulation, 144-fold higher as compared to an extraction of *M. pudica* using ultrasonic cleaner [50], which was about 4.2 mg QE/g only. This might be due to the differences in the extraction techniques employed, where the former incorporated a chemical ethanol extraction with optimal multi-variable interactions, while the latter used a physical ultrasonic approach. Similar to Ganesan et al. [26], the recovery of TFC from microwave-assisted extraction of *M. pudica* resulted in only 61.76 mg RU/g. A high temperature and radiation energy by microwave treatment might disrupt certain bioactive compounds, hence the low recovery of compounds of interest [51].

### 3.2. Correlation Analysis

Based on Table 3, the correlation values of IC_50_ value to TPC and TFC were −0.743 and −0.600, respectively. It can be deduced that the phenolic compounds, which are the predominant components in the *M. pudica* extracts, contributed to the low IC_50_ value. A low IC_50_ value indicates a high accumulation of antioxidative compounds as a low concentration of the extract is sufficient to scavenge at least half of the DPPH free radical. Patro et al. [32] revealed that ethyl acetate extract of *M. pudica* showed a high level of total phenolic and flavonoid contents, and exhibited a significant linear relationship with its antioxidant activity, which was conformed with the present study. The solvent concentration of ethanol had similar impacts to TPC and TFC. Both TPC and TFC recovery in the extracts decreased with increasing polarity of the solvent used. These results reflect those of Sedraoui et al. [52], who also reported that ethanol concentration, solvent-to-sample ratio, and temperature were among the significant (*p* < 0.05) factors affecting the antioxidants extraction from *Phoenix dactylifera*, a date palm species.

### 3.3. Verification of Quadratic Model

**Figure 5:**
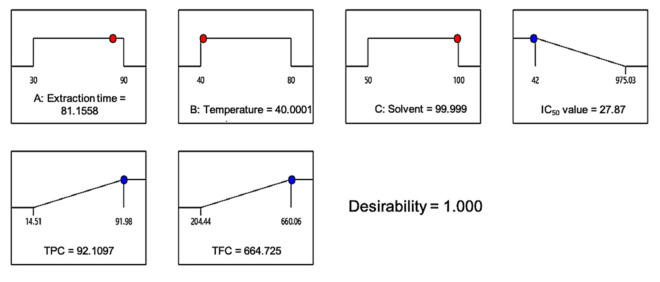
Desirability ramp for numerical optimization of the IC_50_ value and total phenolic and total flavonoid content.

Design-Expert software was used in this study to search for a set of factor levels that satisfied all of the requirements placed on each of the factors and responses. The optimization goals (i.e., none, maximum, minimum, target, or in range) were set for all factors and responses in which the desirability value was obtained. To acquire a good set of conditions with high desirability value, the three variables: (i) extraction time, (ii) temperature, and (iii) ethanol concentration, were set within range while the responses; IC_50_ values, were set at the minimum; and TPC and TFC were set at the maximum. Since extract yield was negatively correlated with the accumulation of the antioxidative compound, it was omitted in the verification part, in order to achieve the main objective of this study. The “importance” of goals for all variables and responses were considered to be equally important and set at 5. The desirability ramps that were developed from optimum points via numerical optimization are exhibited in Figure 5.

To validate the generated quadratic models, a triplicate experiment was employed using the predetermined factor levels. Based on Figure 5, the predicted optimum level of factors (1.000 desirability value) with the condition of 82 min extraction time, 40 °C temperature, and 100% ethanol concentration would yield 27.87 µg/mL of IC50 value, 92.11 mg GAE/g of TPC, and 664.73 mg QE/g of TFC. In the actual experiment, the IC_50_ value obtained was 51.02 µg/mL, while the TPC and TFC obtained were 68.5 mg GAE/g and 659.85 mg QE/g, respectively. The high similarity percentage between the actual and predicted responses indicated that the regression model was suitable and relevant as a reference (Table 4).

### 3.4. Effect of the Optimized M. pudica Extracts on 3T3-L1 Cell Viability

**Figure 6:**
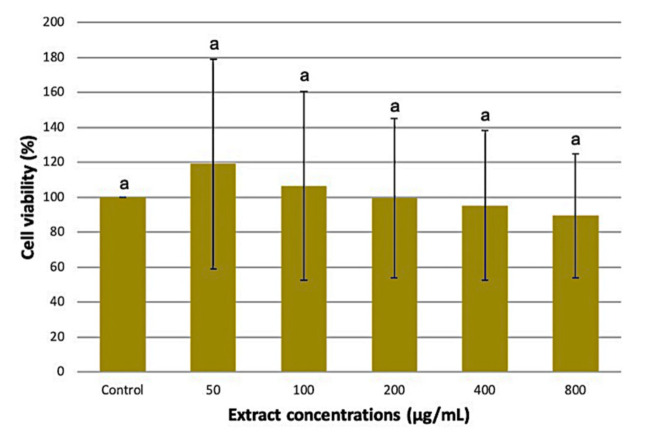
Effects of increasing concentrations of *M. pudica* extract on 3T3-L1 cell lines. Values are expressed as mean ± SE (*n* = 3). Different letters indicate statistically significant differences between factors (one-way ANOVA + Tukey post-hoc test at *p* < 0.05).

MTT assay is a sensitive colourimetric method that assesses the cytotoxic effect of the extracts on cell viability. It measures the reduction of yellow-colored MTT by mitochondrial succinate dehydrogenase into an insoluble dark purple formazan product. The optimized *M. pudica* extracts recovered from the previous experiment were tested on 3T3-L1 preadipocytes to assess their cytotoxicity level at different concentrations (50, 100, 200, 400, and 800 µg/mL). The cell viability results in Figure 6 recorded a non-significant difference between the treated cells and the control (*p* > 0.05). These findings suggest that the optimized *M. pudica* ethanolic extracts caused no harm to 3T3-L1 cells even at relatively high concentrations. On the contrary, *M. pudica* ethanolic extract was found to be cytotoxic to Dalton’s ascites lymphoma cells at an IC_50_ value of 90.33 µg/mL [53] while the methanolic extract of *M. pudica* was cytotoxic to the Rhabdomyosarcoma cell line with a CC_50_ value of 2.03 µg/mL [47]. Since the optimized *M. pudica* extracts were biocompatible to the 3T3-L1 cells, they were subsequently evaluated for glucose uptake assay using 50 and 100 µg/mL extract concentrations.

### 3.5. Effects of the Optimized M. Pudica Extracts on Stimulating Glucose Uptake in 3T3-L1 Adipocyte Cells

**Figure 7:**
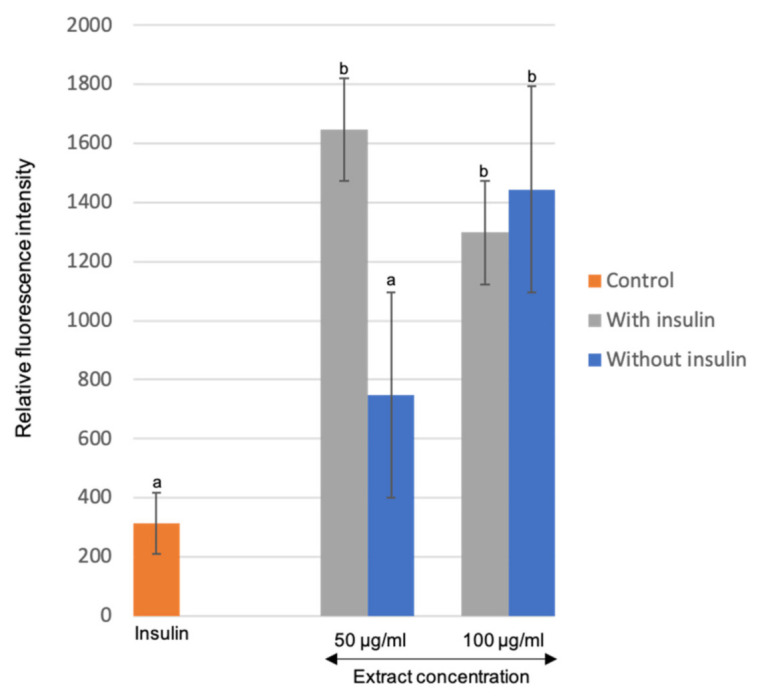
Effects of the *M. pudica* extracts on glucose uptake by 3T3-L1 matured adipocyte cells. Values are expressed as mean ± SE (*n* = 3). Different letters indicate statistically significant differences between factors (one-way ANOVA + Tukey post-hoc test at *p* < 0.05).

A preliminary antidiabetic capacity of *M. pudica* extracts was assessed on matured adipocytes of the 3T3-L1 cell line using 2-NBDG as a marker for glucose metabolism. The increase in relative fluorescence intensity (rfi) exhibited the elevation of 2-NBDG absorption into cells, indicating that the extracts may have insulin-mimetic characteristics [48]. Based on Figure 7, cells treated with only insulin (0.58 mg/mL) were prepared as the reference treatment and recorded at 314.0 rfi. For treatment of extracts alone without insulin, the rfi recorded were 748.67 and 1444.33 for 50 μg/mL and 100 μg/mL of extracts, respectively. The rfi was significantly increased for the treatment of extracts in the presence of insulin, with 1646.0 rfi for 50 μg/mL and 1297.67 rfi for 100 μg/mL of extracts. Surprisingly, the concoction between *M. pudica* extracts and insulin exhibited a synergistic effect, where the glucose uptake rate of the extracts with insulin doubled as compared to extracts alone, and six-fold as compared to insulin alone. There was a significant difference between the glucose uptake rate in the treatment of extracts with insulin and without insulin (*p* < 0.05). Although a higher concentration of extracts (100 µg/mL) combined with insulin exhibited a marginally lower rate of glucose uptake as compared to 50 µg/mL, but there was no significant difference in relation to the concentrations of extract (*p* > 0.05).

Insulin is a hormone that controls blood glucose levels by triggering a variety of physiological reactions in the tissues it targets. It facilitates glucose absorption from the blood by promoting membrane trafficking of the glucose transporter GLUT4 from GLUT4 storage vesicles to the plasma membrane in adipose tissue and skeletal muscle [54]. Insulin stimulation of adipose tissue for glucose absorption is essential for decreasing postprandial blood glucose levels. One of the key causes in the development of type 2 diabetes is abnormal control of this mechanism. A study revealed that *M. pudica* seeds had an antidiabetic impact via increasing pancreatic insulin and α-amylase production, as well as flavonoids and phenols, where it significantly decreased lipid peroxidation and enhanced endogenous antioxidant levels [55]. Another study discovered that methanolic extract of *M. pudica* demonstrated substantial antidiabetic and antihyperlipidemic effects on streptozotocin-induced diabetes mellitus in rats [56]. The antidiabetic potential of the methanolic *M. pudica* extract is equivalent to that of glibenclamide, as indicated by the normalization of blood glucose level tests. Furthermore, Rajendiran et al. [57] and Yupparach and Konsue [58] revealed that ethanolic extract of *M. pudica* leaves exhibited its maximal antidiabetic potential at a dose of 300 mg/kg/bwt against a high-fat diet and streptozotocin-induced type 2 diabetes mellitus rats.

Glucose uptake stimulation assays on 3T3-L1 cells have been performed extensively on various plant species but none of them used *M. pudica*. A study conducted on the extracts of *Sclerocarya birrea* and *Ziziphus mucronata* at a concentration range from 1.56 to 6.25 μg/mL significantly enhanced the glucose uptake in 3T3-L1 cells, which was much greater than that demonstrated by insulin at 6.25 μg/mL [48]. Likewise, the extracts of *Coix lachryma-jobi* exhibited a similar glucose uptake stimulation on 3T3-L1 cells at 30 µg/mL, which was four-fold higher than the basal level and just slightly lower than the insulin at 200 nM [59]. The findings of the present study inferred that *M. pudica* may have hypoglycemic effects and antidiabetic properties that mimic insulin activity. This is the first report to demonstrate an increased glucose uptake in adipocyte cells induced by the ethanolic extracts of *M. pudica*.

## 4. Conclusions

Herbal medicine has long been used in many cultures across the world as a cost-effective therapeutic alternative. In this study, an RSM-based optimization was successfully employed to obtain the optimum extraction conditions of *M. pudica* while maximizing the accumulation of antioxidant compounds. The optimized extraction conditions achieved in this study were 82 min (extraction time), 40 °C (temperature), and 100% ethanol (solvent concentration). The lowest IC_50_ value recorded was 42.0 µg/mL using 100% ethanol as compared to 975.03 µg/mL using 50% ethanol. The highest TPC and TFC value recorded was 94.015 mg GAE/g and 1320.13 mg QE/g using 100% ethanol, which was five-fold and three-fold higher as compared to using 50% and 75% ethanol, respectively. It was found that crude extract yield was inversely proportional to TPC and TFC. The results indicated that ethanol concentration was the most significant factor, having the greatest impact on the accumulation of antioxidant compounds in the extract. The cell viability results recorded a viability level of above 50% for all treatments. The glucose uptake in 3T3-L1 cells treated with 50 μg/mL extract combined with insulin was five-fold higher than the control.

The results of the present work suggest that there is a possible correlation between antioxidants’ accumulation in *M. pudica* extracts and its antidiabetic effects. The high level of free radical scavenging activity, together with the ability to stimulate glucose uptake in adipocyte cells, indicated that *M. pudica* could be a potential biocompatible candidate of a herbal supplement containing active ingredients, which could fight against free-radical-associated oxidative damage, and hence reduce the detrimental effects of diabetes. To confirm and elucidate the mechanism behind this action, further research on the bioactive profile of the optimized ethanolic *M. pudica* extracts should be conducted in the future.

## Figures and Tables

**Table 1:** The values of factors and responses for extraction optimization of *M. pudica* in BBD.

Run	Extraction Time (min)	Temperature (°C)	Ethanol (%)	Extract Yield (%)	DPPH (IC_50_, µg/mL)	Phenolic Content (mg GAE/g)	Flavonoid Content (mg QE/g)
	A	B	C	Y_1_	Y_2_	Y_3_	Y_4_
1	30	60	100	4.0	72.0	76.03	606.31
2	30	60	50	25.9	975.03	14.51	209.44
3	60	40	100	5.0	42.0	85.87	660.06
4	30	80	75	17.0	424.0	62.79	415.69
5	60	60	75	13.6	106.5	42.39	260.69
6	60	80	100	7.3	45.0	80.07	476.31
7	90	40	75	15.4	73.2	68.9	385.69
8	60	60	75	23.1	283.5	59.06	436.31
9	60	60	75	13.9	58.67	65.42	400.06
10	90	60	100	3.1	51.5	91.98	574.44
11	60	80	50	14.6	689.0	20.82	357.56
12	60	60	75	16.2	82.5	69.96	490.06
13	60	60	75	14.0	100.2	49.66	408.81
14	30	40	75	17.3	170.5	34.36	204.44
15	90	60	50	17.9	819.92	31.38	214.44
16	90	80	75	21.5	358.0	70.52	335.06
17	60	40	50	18.6	220.0	39.71	341.94

**Table 2:** ANOVA results of BBD.

		**Extract Yield**				**IC_50_ Value**			
**Sources**	**df**	**Sum of Squares**	**Mean Square**	***F*-Value**	***p*-Value**	**Sum of Squares**	**Mean Square**	***F*-Value**	***p*-Value**
Model	9	563.34	62.59	3.67	0.0501	1.259 × 10^6^	1.574 × 10^5^	10.22	0.0018
A-Extraction time	1	4.96	4.96	0.2912	0.6062	14,357.50	14,357.50	0.9317	0.3627
B-Temperature	1	2.10	2.10	0.1233	0.7358	1.276 × 10^5^	1.276 × 10^5^	8.28	0.0206
C-Ethanol	1	414.72	414.72	24.34	0.0017	7.772 × 10^5^	7.772 × 10^5^	50.43	0.0001
Interaction									
AB	1	10.24	10.24	0.6010	0.4636	*et	*et	*et	*et
AC	1	12.60	12.60	0.7397	0.4182	4529.96	4529.96	0.2939	0.6025
BC	1	9.92	9.92	0.5824	0.4703	54,289.00	54,289.00	3.52	0.0974
Square									
A^2^	1	9.41	9.41	0.5524	0.4815	1.370 × 10^5^	1.370 × 10^5^	8.89	0.0176
B^2^	1	0.0885	0.0885	0.0052	0.9446	10623.70	10,623.70	0.6894	0.4305
C^2^	1	102.34	102.34	6.01	0.0441	1.260 × 10^5^	1.260 × 10^5^	8.17	0.0212
Residual	7	119.26	17.04			1.233 × 10^5^	15,410.79		
Lack of Fit	3	54.77	18.26	1.13	0.4364	91,008.99	22,752.25	2.82	0.1697
Pure Error	4	64.49	16.12			32,277.34	8069.34		
R^2^					0.8253				0.9108
Adequate precision					6.4440				10.4044
		**TPC**				**TFC**			
**Sources**	**df**	**Sum of Squares**	**Mean Square**	***F*-Value**	***p*-Value**	**Sum of Squares**	**Mean Square**	***F*-Value**	***p*-Value**
Model	9	7469.68	829.96	6.24	0.0123	2.369 × 10^5^	29608.63	4.13	0.0304
A-Extraction time	1	705.02	705.02	5.30	0.0548	679.88	679.88	0.0949	0.7659
B-Temperature	1	3.59	3.59	0.0270	0.8741	7.05	7.05	0.0010	0.9757
C-Ethanol	1	6471.01	6471.01	48.6	0.0002	1.781 × 10^5^	1.781 × 10^5^	24.87	0.0011
Interaction									
AB	1	179.69	179.69	1.35	0.2831	17145.28	17145.28	2.39	0.1604
AC	1	0.2116	0.2116	0.0016	0.9693	339.85	339.85	0.0475	0.8330
BC	1	42.84	42.84	0.3222	0.5880	9937.10	9937.10	1.39	0.2727
Square									
A^2^	1	1.77	1.77	0.0133	0.9113	15,736.65	15736.65	2.20	0.1765
B^2^	1	26.18	26.18	0.1969	0.6706	*et	*et	*et	*et
C^2^	1	42.42	42.42	0.3191	0.5898	16,597.46	16597.46	2.32	0.1664
Residual	7	930.61	132.94			57,291.98	7161.50		
Lack of Fit	3	420.62	140.21	1.10	0.4463	28,381.17	7095.29	0.9817	0.5069
Pure Error	4	509.99	127.50			28,910.80	7227.70		
R^2^					0.8892				0.8052
Adequate precision					8.5551				6.6924

*et: excluded terms from the model quadratic equation.

**Table 3:** Correlation analysis between extraction conditions and responses.

	A: Extraction Time	B: Temperature	C: Ethanol Concentration	Crude Extract Yield	IC_50_ Value	TPC	TFC
A: Extraction time							
B: Temperature							
C: Ethanol concentration							
Extract Yield	−0.085	0.055	−0.779		0.612	−0.658	−0.749
IC_50_ value	−0.102	0.304	−0.750	0.612		−0.743	−0.600
TPC	0.290	0.021	0.878	−0.658	−0.743		0.832
TFC	0.048	−0.005	0.778	−0.749	−0.600	0.832	

**Table 4:** Predicted and actual values of responses.

	IC_50_ Value, µg/mL	TPC, mg GAE/g	TFC, mg QE/g
Predicted	27.87	92.11	664.73
Actual	51.02 ± 2.07	68.5 ± 3.15	659.85 ± 31.46
Similarity percentage, %	54.63	74.36	99.27

## Data Availability

Not applicable.
